# Supplementary material for: 3D visualization reveals the cooling rate dependent crystallization near a wall in dense microgel systems
Source: Soft Matter. 2024 Jun 20;20(32):6343–52. doi: 10.1039/d4sm00517a (PMC11323939; doi:10.1039/d4sm00517a)
Supplement: SM-020-D4SM00517A-s001 [file SM-020-D4SM00517A-s001.pdf]

Electronic Supplementary Material (ESI) for Soft Matter.
This journal is © The Royal Society of Chemistry 2024

3D Visualization Reveals Cooling Rate Dependent Crystallization near a Wall in Dense Microgel Systems

ELECTRONIC SUPPLEMENTARY INFORMATION

M.P.M. Schelling, T.W.J. Verouden, T.C.M. Stevens, J.-M. Meijer

Department of Applied Physics and Science Education, Eindhoven University of Technology, P.O. Box 513, 5600 MB Eindhoven, The Netherlands

Institute for Complex Molecular Systems, Eindhoven University of Technology, P.O. Box 513, 5600 MB Eindhoven, The Netherlands

S1 Microgel synthesis and characterization

We used the synthesis procedure reported in [J. Appel et al., *Part. Part. Syst. Charact.*, **32**, 764770 (2015)] [1] to prepare PNIPAM microgels containing a fluorescent core. All chemicals were used as received from the suppliers. For the core synthesis 8.93 g 2,2,2-trifluoroethyl methacrylate (TCI Chemicals, 98%), 0.994 g *N*-isopropyl acrylamide (NIPAM, Merck, 97%), 16.9 mg sodium dodecyl sulfate (Merck, 98.5%), ~ 10 mg Pyrromethene 546 (Merck) and 33 mL water (purified using a MilliQ® Direct 8 system, 18.2 MΩ·cm) were placed in a 100 mL round-bottom flask. The mixture was bubbled with N_2 for 30 min while being heated to 75 °C and stirred at 400 RPM (Fisherbrand Oval PTFE Stir Bar, 25x12mm). The polymerization was initiated by injection of 2.5 mL a 20 mg/mL potassium persulfate (KPS, Merck, 99%) solution, and left for 4 hours (75 °C, 400 RPM stirring). Finally, the latex suspension was filtered to remove any coagulum.

Microgel shells were obtained via seeded precipitation polymerization. 1.195 g NIPAM, 17.4 mg *N,N'*-methylenebisacrylamide (Merck, 99%) (1.0 mol% of monomer), 66.7 µL methacrylic acid (Merck, 99%) (6.9 mol% of monomer), 1 mL core suspension (21.5 wt%) and 50 mL water were placed into a 100 mL round-bottom flask. Again, the mixture was bubbled with N_2 for 30 min while being heated to 75 °C and stirred at 400 RPM. 1 mL KPS solution (51 mg/mL water) was added to start the polymerization, and the mixture was left for 4 hours (75 °C, 400 RPM stirring). The microgel suspension was filtered, and cleaned by repeated centrifugation and redispersion in MilliQ water.

The hydrodynamic size of the microgels was determined with dynamic light scattering using an Anton Paar Litesizer 500 (658 nm, 90 °). Further, the ζ-potential was determined with electrophoretic light scattering using the same device. Samples were prepared at 0.02 wt% in 10 mM NaCl solution. The results of the measurements are given in Supplementary Fig. 1.

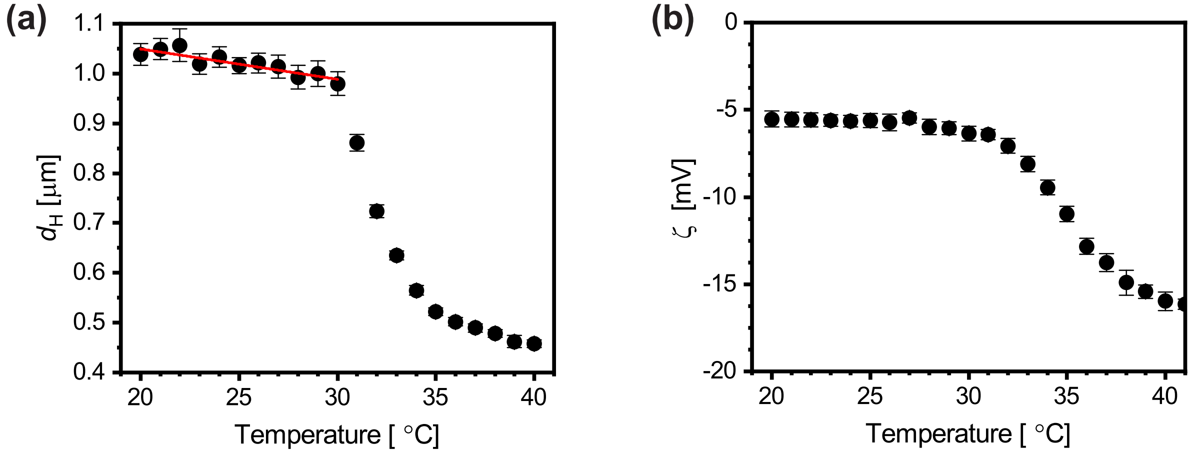

Supplementary Fig. 1 (a) Hydrodynamic diameter d_{H} of the core-shell microgels as function of temperature. Error bars denote the 95 % confidence interval computed from the standard error of the mean (typically from 8 measurements). The red line is a linear fit to indicate the small size change before the Volume Phase Transition. (b) ζ-potential the core-shell microgels as function of temperature. Error bars denote one standard deviation.

S2 Temperature-controlled CLSM sample cell

A schematic illustration of the sample cell used for the experiments is given in Supplementary Fig. 2. The glass ring has an inner diameter of 5 mm and height of approximately 2 mm. The VAHEAT substrate, glass ring and coverslips are glued together using UV glue (Norland Optical Adhesive 68). The objective collar is kept at a constant temperature of 15 °C to allow for fast cooling close to room temperature.

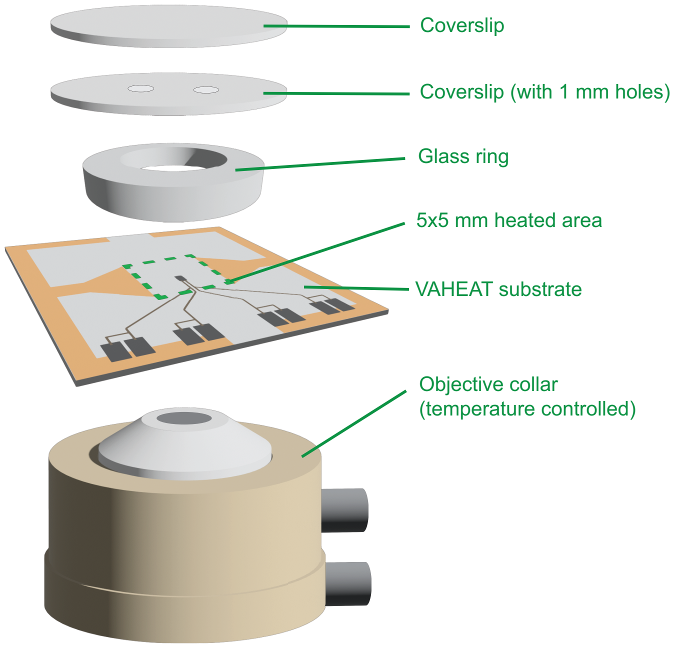

Supplementary Fig. 2 Schematic illustration of the sample cell used for the temperature-controlled CLSM experiments.

S3 Melting point of the dense microgel suspension

To find the melting temperature of the microgel suspension, the sample was heated manually from 20.0 to 28.0 °C in steps of 1.0 °C. Before the heating experiment, the sample was left at room temperature for 2 months, resulting in a crystallized microgel suspension with hexagonally-packed planes aligned with the coverslip. At each temperature, CLSM xyt-scans (120 s, 15.3 fps) were acquired at a distance of approximately 3 μm from the coverslip (corresponding to the fourth layer including the microgels stuck to the coverslip). The xyt-scans were acquired in a 32x32 μm (0.063 μm/px) field-of-view containing around $2 \cdot 10^3$ microgels. From the located microgels, the mean squared displacement

$$\langle \Delta r^2 \rangle(\tau) = \langle |\mathbf{r}(t+\tau) - \mathbf{r}(t)|^2 \rangle \tag{S1}$$

was calculated, where $\mathbf{r} = (x, y)$ is the microgel position, τ is the lag time, and $\langle ... \rangle$ denotes the average over time t and all detected microgels. Supplementary Fig. 3(a) shows $\langle \Delta r^2 \rangle(\tau)$ for temperatures between 20.0 to 28.0 °C. At low temperatures, $\langle \Delta r^2 \rangle$ displays a plateau, as the microgels remain on their lattice site. At elevated temperatures, $\langle \Delta r^2 \rangle$ increases with τ due to long-time diffusion of the microgels, indicating a fluid phase. This transition from crystal to fluid can be clearly visualized by fitting a power law

$$\langle \Delta r^2 \rangle(\tau) = C\tau^{\alpha}, \tag{S2}$$

where C and α are fitting parameters. For Brownian diffusion $\alpha = 1$, while for subdiffusive motion $0 < \alpha < 1$ (e.g. due to caging). The fitted values for α are given in Supplementary Fig. 3(b) and clearly display a transition around 24-25 °C from a solid with near-zero values for α, to a fluid with values close to $\alpha = 1$.

Furthermore, instead of considering the dynamical behavior of the microgels (i.e. their mean squared displacement), the melting point was determined by investigating the degree of order during heating from 20.0 to 28.0 °C. To this end, we define the weighted bond-orientational order parameter[2–4] for each microgel n as

$$\psi_6^*(n) = \frac{1}{\sum_{m=1}^{N} w_{nm}} \cdot \sum_{m=1}^{N} w_{nm} e^{6i\theta_{nm}}, \tag{S3}$$

where N is the number of nearest neighbors m of microgel n, and θ_{nm} is the angle between the vector from microgel n to nearest neighbor m and a reference axis. Furthermore, w_{nm} is a weight proportional to the length the Voronoi cell edge. By averaging $|\psi_6^*|$ over all detected microgels, we obtain the global bond-orientational order parameter $\langle |\psi_6^*| \rangle$, which indicates the degree of hexatic ordering. For a crystal with perfect hexagonal packing $\langle |\psi_6^*| \rangle = 1$, while $\langle |\psi_6^*| \rangle < 1$ implies that hexatic order is lost. Supplementary Fig. 3(b) shows $\langle |\psi_6^*| \rangle$ as function of temperature. Indeed, we find that $\langle |\psi_6^*| \rangle$ sharply decreases around 24-25 °C, indicating a transition from a hexagonally-packed crystal to a fluid.

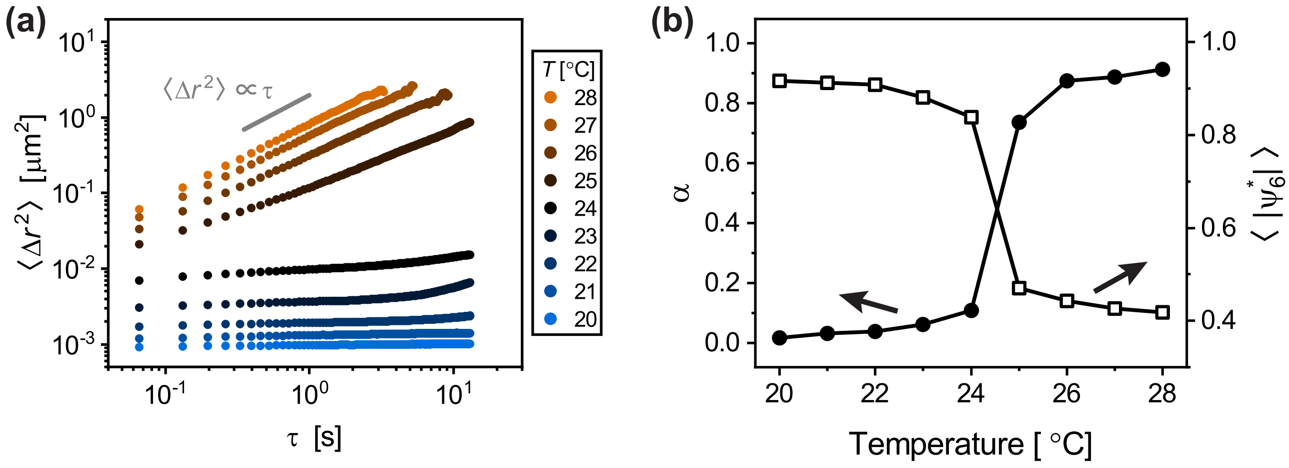

Supplementary Fig. 3 (a) Mean squared displacement $\langle \Delta r^2 \rangle$ as function of lag time τ between 20.0 and 28.0 °C. (b) Left axis, solid circles: α obtained by fitting the data in (a) using Eq. (S2) for $\tau < 3$ s. Right axis, open squares: $\langle |\psi_6^*| \rangle$ as function of temperature.

S4 Sample temperature after quench

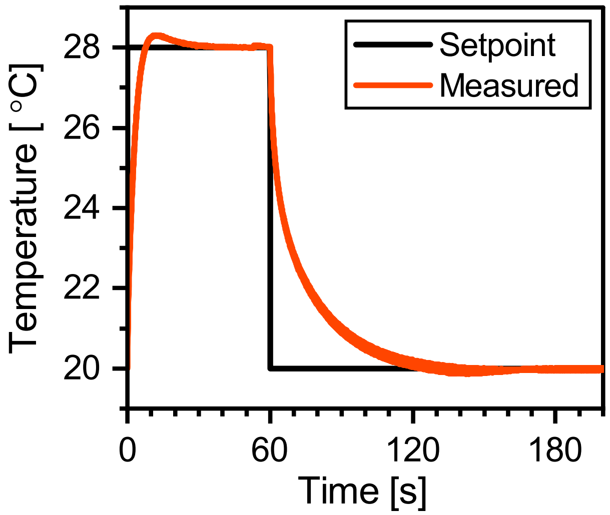

Supplementary Fig. 4 Comparison between the set point temperature and the measured temperatures for the case of the rapid temperature quench. The colored line consists of three overlapping lines corresponding to the three runs.

S5 Determination of HCP and FCC structures with PTM

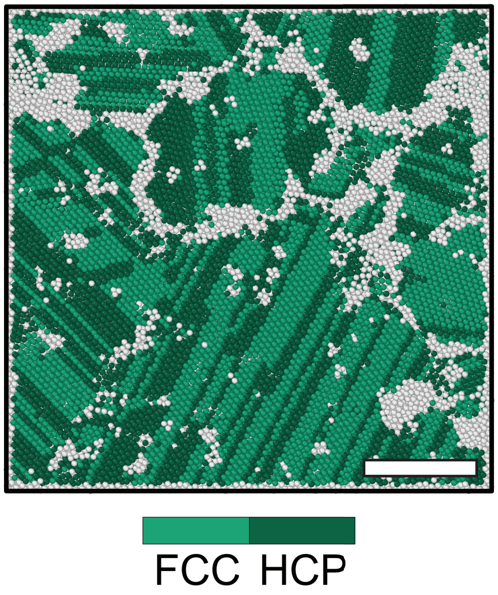

Supplementary Fig. 5 Slice of a 3D rendering (6-8 μm from coverslip) during the slow cooling ramp (squares in Fig. 5(a); 5535 s after the start of the ramp). Colored particles denote those identified as FCC or HCP using PTM[5,6] (RMSD < 0.2); white particles are assigned to neither FCC nor HCP. Scale bar is 15 μm.

References

1 J. Appel, N. De Lange, H. M. Van Der Kooij, T. Van De Laar, J. B. Ten Hove, T. E. Kodger and J. Sprakel, *Particle and Particle Systems Characterization*, 2015, **32**, 764–770.

2 V. Ramasubramani, B. D. Dice, E. S. Harper, M. P. Spellings, J. A. Anderson and S. C. Glotzer, *Computer Physics Communications*, 2020, **254**, 107275.

3 W. Mickel, S. C. Kapfer, G. E. Schröder-Turk and K. Mecke, *The Journal of Chemical Physics*, 2013, **138**, 044501.

4 V. Lotito and T. Zambelli, *Advances in Colloid and Interface Science*, 2020, **284**, 102252.

5 A. Stukowski, *Modelling and Simulation in Materials Science and Engineering*, 2010, **18**, 015012.

6 P. M. Larsen, S. Schmidt and J. Schiøtz, *Modelling and Simulation in Materials Science and Engineering*, 2016, **24**, 055007.
